# Parvovirus Infection and Thrombotic Thrombocytopenic Purpura in an Adult Patient With Sickle Cell Beta-Thalassemia

**DOI:** 10.7759/cureus.16173

**Published:** 2021-07-04

**Authors:** Likhita Shaik, Shaheryar Ranjha, Renuka Reddy Katta, Rutul Shah, Shruti Nelekar

**Affiliations:** 1 Cardiovascular Diseases, Mayo Clinic, Rochester, USA; 2 Internal Medicine, Ahktar Saeed Medical College, Lahore, PAK; 3 internal Medicine, Mayo Clinic, Rochester, USA; 4 Internal Medicine, M. P. Shah Government Medical College, Jamnagar, IND; 5 Internal Medicine, Dr. Vaishampayan Memorial Govt Medical College, Solapur, IND

**Keywords:** parvovirus b-19, atypical ttp, sickle cell beta-thalassemia, vaso occlusive crisis, itp management

## Abstract

Parvovirus infection and thrombotic thrombocytopenic purpura (TTP) are rare manifestations in adults with sickle cell beta-thalassemia. Due to the lack of a clear demarcation between the complications related to sickle cell disease (SCD) and TTP, the diagnosis is often challenging. The treatment requirements for both these entities are divergent and complicated, thus necessitating a careful plan of action during atypical presentations. Here we present a case of a 22-year-old woman during the peripartum period with fever, generalized body aches, and large joint pains that soon evolved into labor. The patient’s history was suggestive of an undiagnosed and inherited blood disorder. The presentation of aplastic crisis-splenic sequestration during early adulthood is atypical for the SCD course in general populations. Moreover, as the patient’s clinical status deteriorated with blood transfusion, the diagnosis and management of a sickle cell crisis event and TTP added to the dilemma in the presence of non-classic parvovirus infection. Though the causation of TTP due to SCD-parvovirus infection is questionable, the treatment of the baseline sickle cell crisis with the novel supportive measures resolved the underlying complications in our patient, suggesting the causal effect. As a result of this, we emphasize the importance of being vigilant about such atypical presentations to avoid delays in diagnosis and treatment of such life-threatening emergencies like TTP.

## Introduction

Sickle cell beta-thalassemia (Hb S/β Th) is a genetic form of sickle cell disease (SCD) that impairs the production of normal hemoglobin (Hb). It results from mutations on the beta (β)-globin gene of Hb that causes polymerization (SCD component) and decreased (β+) or absent (β0) production of β globin chains in the Hb molecule (thalassemia component) [1]. These changes in the structure of hemoglobin form sickled red blood cells (RBC) that have an impaired function, leading to complications. Complications like dactylitis, acute chest syndrome, stroke, and pulmonary hypertension are majorly due to vaso-occlusive (VO) events that follow the sickling phenomenon and compensatory mechanisms to overcome the reduced hemoglobin content in the blood. The coexistence of these two hemoglobinopathies (Hb S/β Th) triggers complications that are also fatal. The diagnosis and treatment for such episodes have been well-known [2-4]. However, certain isolated conditions like idiopathic thrombocytopenic purpura (ITP), thrombotic thrombocytopenic purpura (TTP), and disseminated intravascular coagulation (DIC) have a similar presentation to these complications, hence making the treatment challenging in Hb S/β patients. Moreover, the atypical presentation of such conditions due to lesser-known causative agents poses a clinical and diagnostic dilemma that is challenging to manage [5].

Here we describe a case of a young female in her peripartum period with an undiagnosed Hb S/β Th. She presented with a pain crisis and progressive pathological findings of hemolytic anemia, ITP, and TTP. The atypical nature of the presentation, etiology, laboratory patterns, and management strategy in the setting of Hb S/β Th are the key highlights of our case.

## Case presentation

A 22-year-old gravida 2 para 1 patient of southeast Asian descent came to the rural antenatal care (ANC) unit at 36 weeks of gestation with complaints of fever, severe fatigue, generalized body aches, joint pains (shoulders and knees), and bleeding per vaginum. Her previous pregnancy was complicated by intrauterine growth restriction, a requirement of blood transfusion during labor, and preterm birth at 33 weeks. Past history was significant for multiple episodes of epistaxis that required nasal packing. Family history revealed serial blood transfusions in her mother and sister. Her current admission to the hospital soon evolved into spontaneous labor. The labor was complicated by spiking temperatures, cephalopelvic disproportion (CPD), fetal distress, and maternal fatigue, for which cesarean section was opted for under the cover of penicillin. The patient continued to bleed during the last stage of labor for which uterine vessel cauterization was done. One unit of whole blood was transfused during the labor. She delivered a 1988 g female infant with APGAR (Appearance, Pulse, Grimace, Activity, and Respiration) scores 7 and 8. The rest of the labor and hospital course on Day 1 (day count from hospital admission) was uneventful.

The patient continued to spike temperatures of 101.5 F (vitals: blood pressure: 128/96 mmHg; a heart rate of 86 bpm) on Day 2, which mildly improved with acetaminophen but returned quickly to 101 F after six hours. The physical examination was significant for pallor and mild hepato-splenomegaly. Her blood counts and clinical status as compared to the baseline were deteriorating.

**Table 1:** Laboratory value trends LDH: lactate dehydrogenase; AST: aspartate aminotransferase; ALT: alanine aminotransferase; ALP: alkaline phosphatase

Test	Baseline (before admission)	Day 1	Day 3	Day 5	Day 12
Hemoglobin (g/dL)	10.6	6.1	5.8	5	6.2
Red blood cells (millions/cumm)	2.9	2.23	2.15	2.27	2.8
White blood cells (cells/cumm)	9500	10000	56900	57000	14000
Mean corpuscular volume (fL)	96	91.5	92.7	92.1	92.4
Red cell distribution width (%)	15.2	32.7	35.4	33.5	33.8
Platelets (cells/cumm)	160000	103000	97000	95000	12000
LDH (U/L)	210	250	280	310	260
Haptoglobin (mg/dL)	125	110	80	45	65
Total bilirubin (mg/dL)	1.6	2.1	3.2	5.6	2.0
Indirect bilirubin (mg/dL)	0.9	1.6	1.9	4	1.2
AST (U/L)	40	58	149	112	52
ALT (U/L)	49	76	195	152	94
ALP (U/L)	44	46	58	48	43
Creatinine (mg/dL)	0.98	1.5	1.6	1.2	1.02
Urea (mg/dL)	19	24	48	37	12
Bleeding time (minutes)	7.6	8.5	-	-	7.9
Prothrombin time (seconds)	13.10	13.13	-	-	13.2
International normalized ratio	0.97	0.96	-	-	0.98
Activated partial thromboplastin time (seconds)	40	49.54	-	-	38

**Figure 1:**
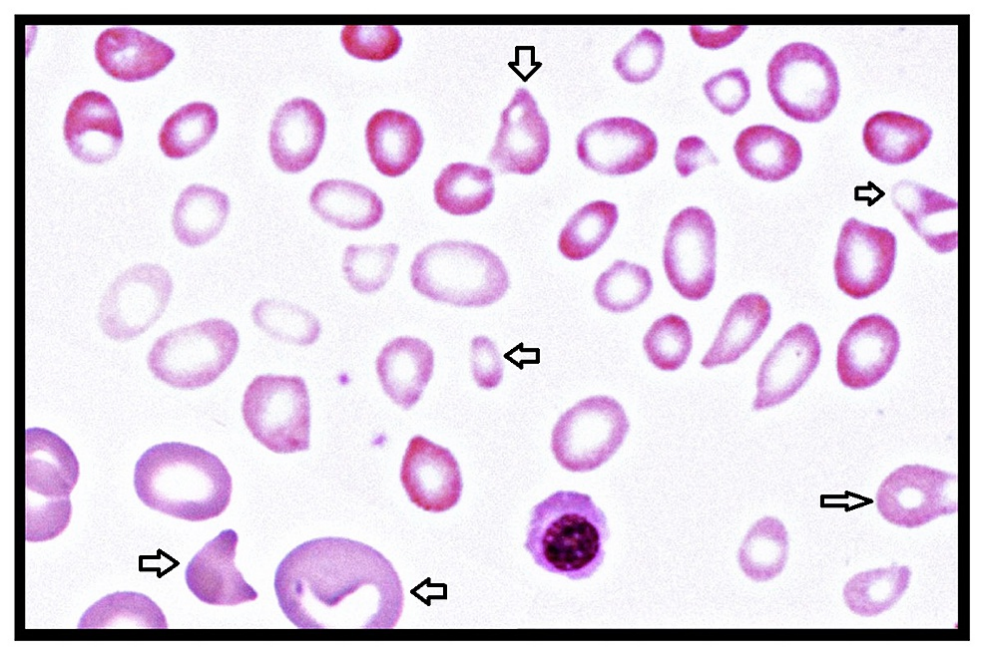
Peripheral smear showing schistocytes and anisopoikilocytosis

**Figure 2:**
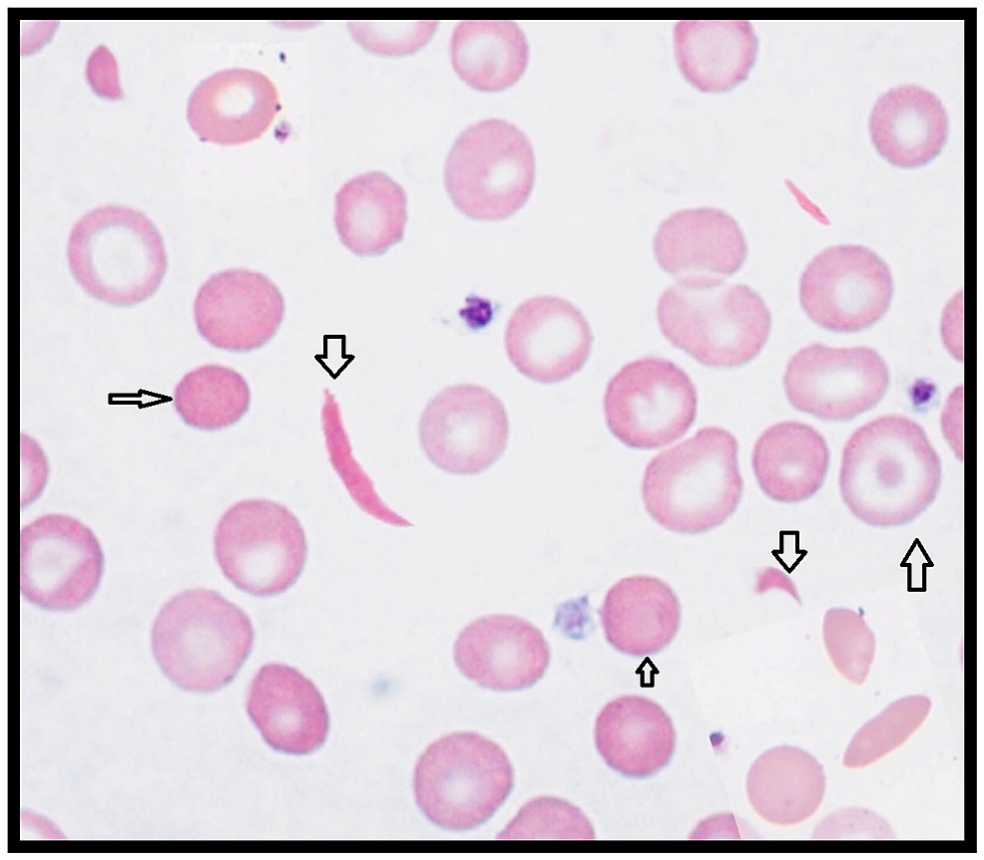
Peripheral smear showing sickle cells, polychromasia, and target cells

Laboratory analysis on Day 2 showed iron deficiency anemia with thrombocytopenia. Transfusion medicine was consulted. Three units of packed red blood cells (p-RBCs) and four units of platelets rich plasma (PRP) were transfused after type-specific and antibody screen. However, her hemoglobin and platelets continued to drop. The patient gradually developed jaundice by Day 3, which was confirmed by high direct bilirubin levels, which peaked on Day 5. The blood counts, liver function tests, lactate dehydrogenase (LDH), haptoglobin values pointed towards hemolytic anemia. Direct Coombs test (DCT) and indirect Coombs test (IDCT), antinuclear antibodies, sickling, osmotic fragility, and glucose-6-phosphate dehydrogenase (G6PD) deficiency testing were negative. On the fourth day, the patient was delirious, with a temperature of 102.2 F. Her joint pains worsened. She also developed dyspnea with a respiratory rate reaching 34/min, mild tachycardia (heart rate ranging between 110 and 120 beats per minute (bpm)). The patient was also screened for the presence of various known pathogens (malaria, Babesia) immunogenic red blood cell antigens (Lea, S, K, Fyb). Serial blood cultures for pathogens like *Staphylococcus, Streptococcus, Enterococcus, Klebsiella, Pseudomonas*, etc. were consistently negative. Physical examination on the fifth day revealed icterus, pallor, and moderate hepato-splenomegaly (HS). Her clinical picture (tachycardia, raised jugular venous pulse (JVP), respiratory distress, crackles in lower lung fields) worsened on Day 5 (Table 1). Clinical suspicion for TTP was raised. Pregnancy-induced TTP was less likely due to its worsening with blood transfusion. This called for a multidisciplinary decision to stop the transfusion and continue expectant management with antipyretics, analgesics, and hydration. Peripheral smear on Day 5 showed microcytosis, hypochromia, polychromasia, and anisopoikilocytosis with schistocytes and sickle cells (Figures 1-2). Complete blood count (CBC), Coombs, and sickling test were again repeated daily. On Day 5, the sickling test and IDCT were positive and the reticulocyte count was 8%, which was a sign of improved bone marrow response.

A preliminary diagnosis of sIckle cell anemia was made. Hb electrophoresis showed Hb F: 24%, Hb A: 8%, Hb A2: 5%, Hb D: 0, Hb S: 63%, Hb C: 0%, and Hb E: 0%, which confirmed the diagnosis of Hb S/β Th. However, the cause of fever with sterile blood cultures was unknown. Hence, polymerase chain reaction (PCR) testing for common viruses was sent, which was positive. The patient was treated with vitamin B12, folic acid supplements, hydroxyurea, and adequate hydration. Joint pain was treated with opioid analgesics. The improvement in clinical and laboratory values led to the decision to discharge and continue with an outlined outpatient management. One week after discharge, most of her laboratory test values returned to baseline. Both the newborn and her previous child have been referred to hemoglobinopathy screenings.

## Discussion

Sickle cell crisis is the commonest cause of hospitalization in SCD. Its components include acute conditions like VO crisis (VOC), aplastic crisis (AC), splenic sequestration crisis (SSC), hyperhemolytic crisis, hepatic crisis, and acute chest syndrome. When these components coexist with infections and life-threatening conditions like ITP, TTP, and DIC, the scenario poses a challenge for management during a crisis [6].

Clinical picture: atypical SCC presentation

VOC usually presents as pain in the extremities, back, and chest areas during childhood. Fever may or may not accompany such episodes. Although this pain is likely due to VOC, other life-threatening causes with similar presentation must be evaluated to avoid misattribution to VOC pain. During late childhood, patients undergo SSC, which presents with sudden pallor, weakness, and abdominal pain that is represented by an acute drop in Hb levels, reticulocytopenia, and HS [7]. SSC may sometimes be clinically confused with AC, however, it can be differentiated by the blood counts. AC is characterized by the direct suppression of bone marrow function evidenced by low Hb and reticulocyte counts (vs SCC has high reticulocyte counts). AC is most frequently caused by parvovirus B-19 but can also be caused by other viral infections or medications [8]. Apart from the AC symptoms that occur in childhood, parvovirus presents with acute, symmetrical small joint pains and a flu-like illness in adult patients similar to one seen in our patient. However, the absence of any such prior episodes during childhood and the presentation of generalized body aches and larger joint pains in the light of an undiagnosed Hb S/β Th did not fit into the classic parvovirus infection bracket in our patient. However, to account for the other symptoms, diagnostics can often help confirm the diagnosis [9].

Diagnosis: SCC vs TTP

The different components of SCC are differentiated based on diagnostics and clinical findings. After the diagnosis of Hb S/β Th, the laboratory findings in our patient, initially suggesting aplastic crisis may be due to parvovirus, also showed thrombocytopenia, which is usually diagnosed as ITP (diagnosis of exclusion) in the absence of other known etiology [10]. However, abnormal values of LDH, haptoglobin, bilirubin, and creatinine depict a hemolytic cause of anemia and thrombocytopenia. Other clues like fever and neurological symptoms suggest more life-threatening issues like TTP and DIC [9,11-12]. However, as is well-known that acquired TTP is caused by medications (chemotherapeutic drugs, ticlopidine, clopidogrel, cyclosporine A, hormone therapy, and estrogens), viral infections (EBV, hepatitis B, C, HIV, varicella), and chronic diseases like lupus, rheumatoid arthritis, and cancers. The clinical suspicion towards TTP due to parvovirus is minimal. Moreover, diagnosing TTP amidst the similarities of fever, anemia, nonfocal neurological symptoms, hemolytic anemia, thrombocytopenia, and renal impairment between SCD and TTP is questionable [13]. However, due to the life-threatening nature of the condition, it calls for immediate management. It is usually diagnosed by an ADAMTS13 enzyme activity, which cleaves the von Willebrand factor that is involved in the clotting process. The test is not widely available in developing countries, hence preliminary TTP diagnosis can be made clinically with thrombocytopenia and schistocytes on peripheral smear [14-15].

Treatment: SCC, parvovirus infection, and TTP

Parvovirus infection is a self-limiting illness in pregnant patients and is treated with supportive measures like hydration, analgesics, antipyretics. However, when it manifests as AC, replenishing the inadequate cell lines through blood transfusion may be necessary. Such measures cause fluid overload and deteriorate patients with TTP. In contrast, TTP requires more aggressive management like plasmapheresis, glucocorticoids, vincristine, rituximab, and cyclosporine A [9,11]. Hence, it is challenging to treat such complicated conditions simultaneously due to the different lines of treatment. If observed closely, the course of events in our patient began with a flu-like illness suggesting parvovirus infection, which was followed by ITP/TTP-like syndrome with impending multiorgan failure in the background of SCD. Due to the occurrence of complications from treating each one separately, we recommend that treating the baseline illness with simpler and novel therapies may prove beneficial as seen in our patient [16]. Though the mechanism is unknown, we believe that the treatment of SCD treated the stress-induced SCC and parvovirus infection, which helped the recovery from TTP.

## Conclusions

Parvovirus is among the least known agents that cause TTP, aplastic crisis, and splenic sequestration simultaneously in an adult patient. It may present atypically as an anaplastic crisis during adulthood with generalized body aches and involvement of large joints. Though the association of parvovirus-SCD with TTP has rarely been described in the literature, physicians must be vigilant of such presentations. Our case is one such example that also recommends that aggressive measures are not always a prerequisite to treating conditions (SCC and TTP) with a clinical overlap. Though the mechanism is yet unknown, simple and novel therapies that treat the baseline condition (SCD) improve morbidity and clinical outcomes in such patients.
